# Poverty, protests and pandemics: what can we learn from community resilience?

**DOI:** 10.1177/00812463211047669

**Published:** 2021-12

**Authors:** Rashid Ahmed, Yusuf Mohamed Sayed, Jonathan Nell, Nceba Z Somhlaba, Abdulrazak Karriem

**Affiliations:** 1 University of the Western Cape, South Africa; 2 University of Sussex, UK

The destruction of property, theft and violence that occurred in South Africa, particularly in KZN and Gauteng in the week of 12 July 2021 had a significant impact on the national psyche. As we try to come to terms with the magnitude of the consequences on the political, economic, psychological and social levels, what are the lessons that we can draw from this adversity? This commentary draws on the notion of community resilience to understand what has happened, and how it may provide markers for the future. The commentary begins with a short overview of the notion of community resilience, followed by a discussion of its utility to explain the events as well as lessons for the future.

Resilience research has grown considerably, particularly in the last decade and has shifted from a narrow focus on individual mental health, particularly early in its development, to a more systemic approach. Nonetheless, concerns remain about its usage and it is a highly contested concept that needs to be problematized. Both terms (i.e., community and resilience) remain complex and contested, but the idea that collectives or groups (i.e., communities) have agency to respond to adversity is a useful framework for understanding the recent conflict and violence in South Africa.

Resilience is best defined as the complex interaction of risk and protective processes, and that in the presence of adversity both negative or adaptive outcomes are possible (Masten, 2001; Ungar, 2004; Ungar & Theron, 2020). Community resilience can be defined as the aggregate level of resources communities develop and utilize in relation to risk or adversity (Pfefferbaum & Klomp, 2013) that allow communities to cope, ‘bounce back’ or transform adversity. Community resilience is more than the sum total of individual resilience and refers to processes that emerge through the complex networks of structures, relationships and resources in these communities, but is not reducible to a description of these resources, as is so often the case. For example, collective or community hope is more than the sense of individual hope of the members of a particular community. Drawing on this understanding, the destruction of property, loss of lives, and violence that occurred could be understood as negative outcomes, while the activation of social support and networks, the cultivation of hope, and social action that emerged in response to these events, could be perceived as an adaptive outcome mitigating the situation of adversity.

We focus on three key areas pertaining to resilience that emerged as the events unfolded. The first is the emergence of and attempts at the building of hope. Alongside the many actions that challenged hope, were constant reminders of, and attempts to build hope. The sharing of images and messages best symbolized by that of a wheelchair-bound individual assisting in the cleaning up operations that followed the events is a powerful symbol of hope in the face of adversity and despair. Second, the multiple altruistic actions such as the distribution of food and other necessities by individuals and organizations, and activation of social support was a significant marker of resilience. It was an attempt to transcend the racial polarization and reflected altruism that countered the immediacy, self-gain and destructive behaviour of those solely focused on benefitting from the situation. Third, communities came together in ways that may not have been present for decades and which was an important characteristic of the struggle against Apartheid. While some of the forms that communities took to protect themselves became destructive, the activation of community processes and systems latent for a long while evidenced community capacity to thrive under conditions of adversity. Neighbours immediately started communicating with each other, social support networks became activated, organic leadership structures emerged, and a collective consensus was reached on what was required, building on the proud traditions of collectivist and solidaristic actions against the system of Apartheid.

While the form of the events and their triggers may be specific, local, and perhaps unexpected, the conditions which account for their emergence, and in particular, the deep and structural inequality within and between countries, have been present for many decades. This was illuminated and exacerbated by the COVID-19 pandemic. The causes for such events are rooted in the global neo-liberal agenda which is characterized by cutbacks on social welfare and social spending, an imposed austerity programme that affected the impoverished the most, and reducing of the state’s role in promoting and protecting the public good. Specifically, unemployment, inequality and poverty have reached unprecedented levels, and global income inequality is the highest it has ever been. For example, the latest Gini coefficient of income inequality for South Africa identifies it as the most unequal society in the world (Stiegler & Bouchard, 2020), with this trend evident in many other countries (Dorling, 2015). Further, the cutbacks to essential areas and the privatization of public services and resources essential to well-being such as health, education and social welfare, all contribute to creating conditions for the growing immiseration of the most marginalized and disadvantaged (Shamir, 2010; Udvarhelyi, 2014). Such an agenda creates a fertile ground for populist rhetoric and agitation and which partly resulted in the violence and destruction in July 2021 in South Africa.

The stories of, and the cultivation of hope remains one of the most poignant lessons from these events. It is a testimony to collective human agency, in spite of the daily assault on hope created by the neo-liberal context sketched above. It is a collective and solidaristic hope that transcends and counteracts individual despair and hopelessness, and that has remained in diverse contexts globally, even during the pandemic.

The extent of the community mobilization again is only surprising to the extent where the current neo-liberal context, for a complex set of reasons, constrains working together and social action. The events in South Africa, while not in the same vein of the community mobilization and grassroots democracy seen in the struggle against Apartheid, demonstrate the vibrancy of community organizations, social movements for justice and other forms of civic engagement. The spontaneous and often sporadic and transient nature of these social movements need to be nurtured and cultivated and should include a critical awareness of all forms of structural oppression and exploitation. More importantly, they need to be utilized in a way that not only helps communities to survive or cope, but also to transform and overcome unequal social, political, and economic conditions in society which denies citizens from claiming their rights. This requires building on the resilience and collective action witnessed to fight for the public good and social justice.

One of the earliest descriptions in the literature frames this as the difference between beating the odds (i.e., positive outcome in spite of adversity) and changing the odds (i.e., changing adversity to produce positive outcomes) (Seccombe, 2002). The indications of resilience identified above suggest we might, and must, be able to do both. It suggests that the indicators of community resilience that were evident can be utilized for a common vision of the public good and social justice that transforms social institutions. However, the evidence of community cohesion and collective action needs to be complemented by a conscientization that locates risk within the neo-liberal context sketched above, and the actions that follow unite rather than polarize. It suggests that the hope we witnessed can flourish, and that altruism and support can extend to the challenges of daily living. It is a reminder that all of us need to make our voices heard, unite in our common humanity and act together whenever there is injustice. The power of the neo-liberal agenda needs to be matched by our collective resilience and the tragedy of what unfolded is one reminder about the long road still to be travelled. The indications are that we have the strength and belief to do it.
